# Elucidating miRNA Function in Cancer Biology via the Molecular Genetics’ Toolbox

**DOI:** 10.3390/biomedicines10040915

**Published:** 2022-04-15

**Authors:** Adam Azlan, Yaashini Rajasegaran, Khor Kang Zi, Aliaa Arina Rosli, Mot Yee Yik, Narazah Mohd Yusoff, Olaf Heidenreich, Emmanuel Jairaj Moses

**Affiliations:** 1 Cluster of Regenerative Medicine, Advanced Medical and Dental Institute, Universiti Sains Malaysia, Bertam, Kepala Batas 13200, Pulau Pinang, Malaysia; adamazlan@student.usm.my (A.A.); yaashini@student.usm.my (Y.R.); kangzi@student.usm.my (K.K.Z.); aliaarosli@student.usm.my (A.A.R.); yymot81@gmail.com (M.Y.Y.); narazah@usm.my (N.M.Y.); 2 Northern Institute for Cancer Research, Paul O’Gorman Building, Medical School, Framlington Place, Newcastle upon Tyne NE2 4HH, UK; o.t.heidenreich@prinsesmaximacentrum.nl; 3 Prinses Máxima Centrum Voor Kinderoncologie Heidelberglaan 25, 3584 CS Utrecht, The Netherlands

**Keywords:** microRNA, miRNASponges, antagomir, miRNA mimic, CRISPR/Cas9, dCas9 modifications, epigenetics

## Abstract

Micro-RNA (miRNAs) are short non-coding RNAs of about 18–20 nucleotides in length and are implicated in many cellular processes including proliferation, development, differentiation, apoptosis and cell signaling. Furthermore, it is well known that miRNA expression is frequently dysregulated in many cancers. Therefore, this review will highlight the various mechanisms by which microRNAs are dysregulated in cancer. Further highlights include the abundance of molecular genetics tools that are currently available to study miRNA function as well as their advantages and disadvantages with a special focus on various CRISPR/Cas systems This review provides general workflows and some practical considerations when studying miRNA function thus enabling researchers to make informed decisions in regards to the appropriate molecular genetics tool to be utilized for their experiments.

## 1. MicroRNA: A Brief Introduction

MicroRNAs were first discovered almost three decades ago by Lee et al. (1993) when his research group discovered that the lin-4 transcripts of 22 and 61 nucleotide (nt) bases long, known at that time as short RNA, controlled the expression of a lin-14 protein which is crucial for embryonic development of *C. elegans* [1]. Sequence study of the lin-4 gene revealed that repeat elements located within the 3′-untranslated region (3′-UTR) are complementary to sequences found inside both of the lin-4 transcripts. This group hypothesized that an RNA–RNA hybrid formation may contribute to the downregulation of lin-14 mRNA, although the complementary sequence stretches only 10 nt base long. Through this landmark research, the term microRNA was coined to describe short RNA oligos of approximately 18 to 20 nt long which functions to regulate protein expression at the transcriptome level [2,3,4].

Many cell types depend on the complex system of microRNAs to regulate their normal biological and physiological functions [5]. However, when these networks are dysregulated, normal cells could acquire oncogenic traits which drive conversion into malignant counterparts [6,7]. Among the common mechanisms which facilitate tumorigenicity include amplification of loci, various classes of mutations, epigenetic modifications on microRNA loci and alterations to transcription factors controlling microRNA expression [8,9]. The accumulation of these changes progressively lead to carcinogenesis and over time bring rise to the various hallmarks of cancer which includes conferring selective proliferative advantage, altered stress response, increased vascularization, enhancement of invasion and metastasis, rewiring of metabolic pathways, evasion of immune response and augmentation of the tumor microenvironment [10,11].

In order to elucidate the various effects of microRNA dysregulation in cancers, a wide array of novel technologies in molecular genetics are required. The rapid progress of discoveries in molecular biology has led to innovative applications which are capable of deciphering mechanisms of disease onset and progression with great precision where of late, research related to microRNA regulation in cancers have incorporated various pioneering molecular techniques including miRNA sponges, antisense oligonucleotides (antagomirs) and miRNA mimics [12,13,14].

Moreover, the advent of computational biology has led to the development of various databases and tools allowing for miRNA target analyses [15]. Target identification is crucial for the translation of miRNA potential activity as protein function predicts the implication of miRNA dysregulation in disorders [16,17]. Tools such as miRDB, TargetScan, mirTarBase and mirWalk, which are openly available online with ease of access and user-friendly interfaces, would significantly aid researchers in identifying a probable target for studied miRNA [18,19,20,21].

More recently, CRISPR has emerged as a game-changing tool in this field bolstering a range of derivative Cas proteins which are capable of interrogating and augmenting different microRNA functions in cancers [22,23,24]. This review will focus to detail the use of various cutting-edge molecular genetics technologies in elucidating the microRNA dysregulation of cancers to provide a clearer perspective on the advantages and limitations of each technique as well as a comparison of their utility in individual studies and delineate practical considerations in the implementation of new strategies such as CRISPR [25,26].

## 2. Mechanisms of microRNA Dysregulation in Cancer

Similar to genes, microRNA are also subjected to dysregulation, therefore various mechanisms need to be considered when studying miRNA dysregulation. Amongst them are the amplification of loci which leads to the overexpression of amplified genes, mutation leading to deregulated or loss of function, epigenetic control contributing to gene activation or suppression and the effects of trans activating elements on gene expressional control.

### 2.1. Amplification of Loci

**Figure 1:**
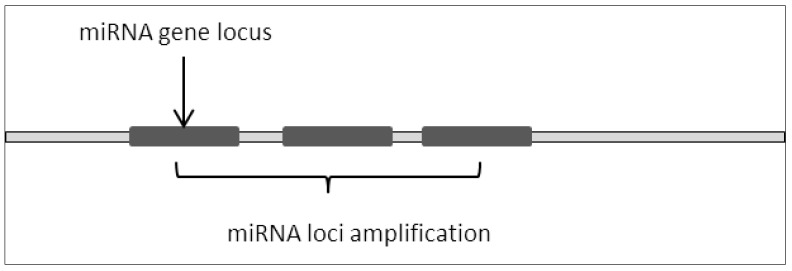
miRNA loci amplification resulted in repeating miRNA sequences thus increasing basal expression of miRNA.

MicroRNA loci amplification is a common occurrence in various cancers (Figure 1). Amplification of the genomic region often causes overexpression of the miRNAs thus resulting in the depletion of its target mRNA [27]. This in turn leads to dysregulation in critical signaling pathways which contribute to cancer development and progression.

This can be observed in embryonic brain tumors where the C19 miRNA cluster was amplified [28,29]. Interestingly, the same research also showed that the C19 cluster was fused to the TTYH1 gene, which further drives the expression of the amplified C19 cluster polycistronically. This causes extreme overexpression of DNM3TB which is an effector of the C19 miRNA cluster target, RBL2. This study clearly shows that microRNA loci amplification leads to the dysregulation of target genes and their effectors.

Amplification also occurred in the miR-17-92 cluster where it was found that the miRNAs within the cluster were significantly overexpressed in human lung cancer [30]. MiR-17-92 cluster copy number was significantly higher when compared to the copy number of miR-106a-92 and miR 106b-25. Additionally, the same cluster was also found to be amplified in acute myeloid leukemia with MLL rearrangement where it was found that this cluster affects cellular differentiation and proliferation [31].

Furthermore, miR-23a locus was also found to be implicated in the same manner in gastric cancer. Research found that the miRNA was over-expressed and that the miRNA gene was amplified along with other miRNAs (miR-1274a, miR-196b, miR-4298, miR-181c, miR-181d, miR-23a, miR-27a and miR-24-2) at loci 19p13.13. The upregulation of these miRNAs contributed to gastric cancer progression. Additionally, the group also revealed that miR-23a downregulated MT2A and that inhibition of the microRNA halted tumor growth [32].

It was also reported that amplification of miR-191/425 promotes the growth of breast cancer [33]. The group reported that the copy number of miR-191/425 was significantly higher in primary breast tumor in comparison to normal tissue. MicroRNA-191/425 target DICER1 which leads to the global miRNA dysregulation due to its function in processing pre-miRNA into mature miRNA, thus effectively leading to the promotion of breast cancer tumorigenesis.

### 2.2. Mutation (Single Point Mutation, Deletion, Insertion, Base Substitution)

**Figure 2:**
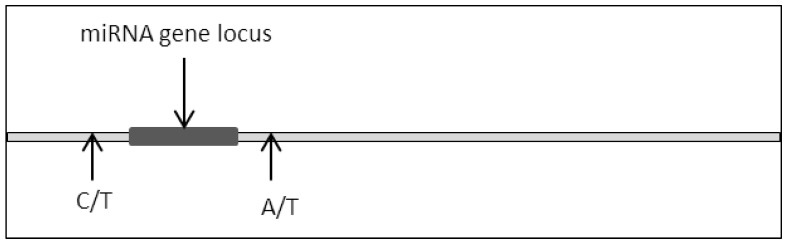
Single base disposition at miRNA locus.

Mutation to genes such as substitution or deletion often leads to the production of nonfunctional translated proteins due to impaired codon arrangements [34,35,36]. As a consequence, critical cellular signaling pathways are frequently dysregulated due to the impairment in protein structure and function where this culminates with the development of various cancers [37,38,39,40]. A similar phenomenon can be observed in miRNA where a mutation in the miRNA locus would lead to dysfunctional miRNA expression (Figure 2).

This was proven in the case of the miR-15a and miR-16-1 locus where partial homozygous deletion of the locus led to the depletion of miR-15a expression. Furthermore, the group also reported a single base substitution of T to C 21bp upstream of the miR-15a coding sequence. It was postulated from this research that these mutations lead to the development of prostate cancer due to the prevalence of these mutations in xenografts [41].

Furthermore, point mutation of miRNA was also found to play a hand in leukemogenesis. It was found that point mutation in the miR-142 resulted in the loss of function of that miRNA. This potentiates AML towards myeloid lineages while suppressing lymphoid lineages differentiation. The loss of miR-142 leads to the accumulation of ASHL1 resulting in the maintenance of the HOXA genes. This caused the myeloid progenitors to be locked in the myeloid stage thus preventing proper cellular differentiation [42]. Additionally, it was also reported from cancer cohort (TCGA) data that the miR-142-3p seed region harbors the mutation at the 7th and 6th nucleotide where A > G and C > G/T occurs predominantly in diffuse large b-cell lymphoma [43]. Earlier results reported also that mutation in the miR-142-3p seed sequence shifted miR-142 targeting from RAC1 towards ZEB2 in diffuse large b-cell lymphoma which could attribute to disease progression [44].

### 2.3. Epigenetic Modifications on miRNA Loci

**Figure 3:**
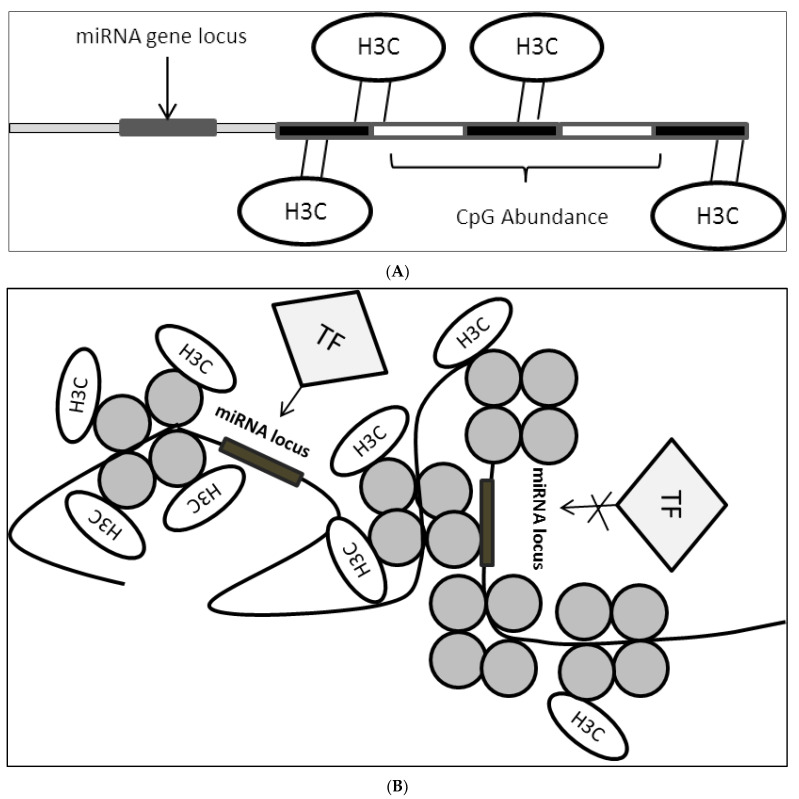
(**A**): CpG site enrichment on miRNA locus resulting in miRNA suppression. CpG methylation brought about by DNMTs methylates DNA sequence in proximity to miRNA locus or direct locus methylation leads to suppression of miRNA expression. H3C: methyl group; (**B**): Histone state on miRNA locus controls accessibility of TFs. Histone modification leads to the formation of open or closed chromatin structure which in turn affect TFs binding onto miRNA locus where this would result in either miRNA activation or suppression depending on the transcriptional activity of the bounded factors. H3C: methyl group.

Epigenetic modulation plays a critical role in the regulation of miRNA expression [45,46]. Epigenetic markers such as DNA methylation, histone state, an abundance of CpG sites and binding of epigenetic modulators influence the accessibility of miRNA loci to various transcription machinery (Figure 3A,B). In this case, a group reported that PPARγ binds consensus sequence located on the miR-122 promoter to promote expression by inducing histone acetylation. They reported that induction of hepatocellular carcinoma cells with 5′aza-2′deoxycytidine (a compound that inhibits methyltransferase) prevented the N-cor/SMRT/HMeT complex, which is a miR-122 inhibitor from binding onto the consensus promoter sequence which in turn caused a reduction in H3K9 demethylation (heterochromatin mark on the genome, usually found to be abundant in repressed genes) and provided an accessible miR-122 locus which allowed PPARγ to bind predominantly [47].

Histone state plays a major role in the accessibility of crucial transcription machinery onto gene locus which triggers transcription or suppression. Depending on the state of chromatin brought about by the actions of histone modifiers genes can either be activated or inactivated. In miRNA regulation, it was reported that the interplay between miRNA and histone modifiers exists in modulating disease progression [48,49,50]. It was found that miR-125a-5p expression negatively regulates HDAC5 in breast cancer which contributes to disease progression via a feedback loop. The study found that miR-125a-5p binds the 3′-UTR of HDAC5 which leads to the reduction in HDAC5 dependent RUNX3 acetylation on p300/RUNX3 complex resulting in the dissociation of the complex binding on the miR-125a-5p locus. Using HDAC inhibitor, HDAC5 activity was suppressed leading to the reduction of miR-125a-5p which leads to apoptosis suggesting interplay between miRNA and HDACs in mediating apoptotic evasion [51]. In addition, it was also reported that the EZH2 histone modifier, which induces heterochromatinization, was overexpressed in pancreatic duct adenocarcinoma (PDAC) leading to the suppression of miR-218. Direct EZH2 siRNA targeting leads to restoration in miR-218 expression as this abrogates EZH2 binding onto the miRNA locus also blocking the recruitment of DNMTs via EZH2 [52].

Additionally, it was also shown that CpG sites, influence the expression of miR-335 located within the MEST gene locus downstream of MEST exon 1 which may contribute to hepatocarcinogenesis. In this study, it was found that the CpG-rich site located upstream of the miR-335 beyond exon 1 of MEST resulted in the repression of miR-335 expression due to DNA hypermethylation. The group also reveals that treatment with 5′aza-2′deoxycytidine, a known DNA demethylator, resulted in the upregulation of the suppressed miR-335 [53].

It was found that in prostate cancer overexpression of the MED1 gene would lead to the disease progression [54,55]. This effect could be mitigated via MED1 suppression by miR-205. However, it was found that the miR-205 locus was hypermethylated in prostate cancer thus leading to the epigenetic silencing of the miRNA. Additionally, the same group found that H3K9Ac, a histone mark for euchromatin (highly accessible areas within the genome usually susceptible to transcription factor binding), was significantly reduced on miR-205 loci. Furthermore, they also reported the prevalence of epigenetic in various cancer cells and found that miR-205 expression was inversely correlated with DNA methylation [56,57].

Results have shown that miR-195 was epigenetically influenced as well. It was found that the expression of miR-195, a tumor suppressor miRNA that is required for normal colon function, was downregulated in colorectal cancer cells [58,59]. It was also shown in the study that CpG islands upstream of the miR-195 locus were partially methylated in normal colorectal cells. However, in colorectal cancer, this site was fully methylated [60]. The findings from these studies suggest that DNA hypermethylation of miRNA loci in colorectal cancer plays a crucial role in oncogenesis by interfering with the tumor-suppressive activity of miR-195.

Reports have also shown that miR-340 is implicated epigenetically in neuroblastoma [61,62]. CpG-rich sites were found downstream of the predicted transcription start site (TSS) of miR-340 and these sites were extensively methylated. Interestingly, these sites were also found to overlap with RNA polymerase II (pol II) binding sites thus suggesting epigenetic modulation in transcriptional control of miRNA [63]. Treatment with 5′aza-2′deoxycytidine reduced methylation of the CpG sites which in turn led to an increase in miR-340 activity via reduction of its target gene SOX2. These findings clearly highlight the role of epigenetic modifications in miRNA dysregulation.

### 2.4. Transcription Factors (TF) Controlling miRNA Expression Dysregulation

Transcription factors (TFs) are well known to either promote or suppress gene expression [64]. Transcription factors are generally understood as proteins that bind specific consensus sequences located on genomic regions, particularly, on gene loci [65,66]. The binding of TFs can either occur proximal or distal from the TSS [67,68]. TFs that promote accessibility on the genomic region for RNA pol II binding and other TFs are known as activators whereas suppressors are TFs that promote inaccessibility [69]. The mechanism of action of TFs involves the recruitment of other TFs or epigenetic modulators on specific genomic loci. Depending on what type of TF bounded (Suppressors or Activators) they partner with different epigenome modulators (HDAC, HMET, HAT, p300). This leads to either upregulation or downregulation of genes affected [70,71].

Proper TF function is crucial for the regulation of miRNA expression. This can clearly be seen in the interaction between the transcription factor ETS5 and miR-155. It was shown that ETS5 binds to miR-155 TSS and activates the expression of the pri-miR-155 which in turn leads to lipopolysaccharide activation in the human embryonic kidney. Contrarily, the expression of miR-155 was downregulated by the addition of IL-10, an inhibitor of ETS5. This is an example of an activator mechanism in miRNA expression [72].

Additionally, it was also found that GATA3 modulates the activity of miR-29b by binding on the promoter of miR-29b [73]. Reporter assay also showed induction in expression when miR-29b loci containing the GATA3 binding sequences were cloned into luciferase plasmids [74]. However, deletion of the promoter binding sequence resulted in the reduction in luciferase activity clearly showing that GATA3 binding is crucial for miR-29b induction.

Interestingly, it was also reported that miR-1 and miR-206 were both regulated by NRF2 transcription factors in an HDAC4-dependent manner [75,76]. In this study, downregulation of NRF2 resulted in an increase in miR-1 and miR-206 expression. This indicates that the TF acts as a suppressor. NRF induces HDAC activity resulting in a reduction in accessibility (heterochromatin) on the genomic loci, thus inactivating transcription. Furthermore, downregulation of HDAC resulted in an increase in miR-1 and miR-206 expression.

A summary of the various mechanisms involved in miRNA dysregulation is presented in Table 1.

## 3. Conventional Molecular Genetics Methods for Studying miRNA Dysfunction

**Figure 4:**
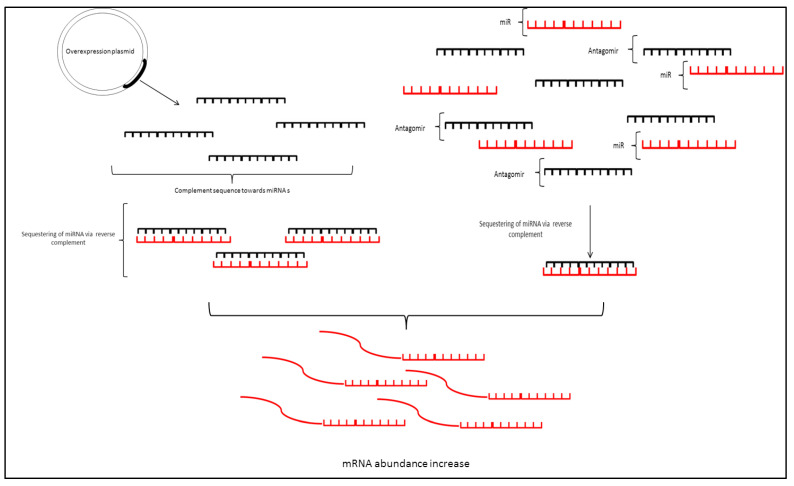
Tools for regulating miRNA; miRNA sponges use overexpression plasmids to produce miRNA target sequence which sequesters miRNAs reducing binding on target mRNA thus increasing target mRNA expression whereas antagomir is a complementary sequence of short RNA that binds mRNA via base pairing, the use of antagomir prevents miRNA from inducing mRNA cleavage thus resulting in increase in target expression.

Currently, a myriad of molecular genetic techniques is available to study the various phenomenon that cause miRNA dysregulation. (Figure 4). The subsequent portions of this review will describe each method in detail.

### 3.1. MicroRNA Sponges

In essence, miR-Sponges sequester miRNAs by overexpressing their target complementary 3′-UTR sequences using a plasmid-based system where the 3′-UTR is expressed using a strong promoter, e.g., the CMV promoter, basically, these plasmids overexpressed the miRNA target sequence in which miRNA binds thus “pull” the miRNAs away from their target sequence [87,88]. The expression of the miRNA target sequences act by providing an accessible template for the target miRNA to bind, this, in turn, will block the miRNA from binding to target messenger RNA as the miRNA has a higher affinity for overexpressed, readily available, target site. This leads to the knockdown effect of targeted miRNAs as sponges sequester miRNA from binding to its actual target, resulting in the accumulation of the mRNA. This system usually fuses the 3′-UTR (miRNA target sequence) at the 3′ end of a reporter gene such as GFP or luciferase [89,90]. A CMV promoter plasmid drives the reporter gene expression bearing the miRNA target sequence on its 3′-UTR constitutively. The sequestering of the targeted miRNAs can be seen via a reduction in reporter gene signal in comparison to control which expresses reporters without the miRNA target sequence.

Studies also showed that endogenous sponges or “natural sponges” act in the same manner where they regulate miRNA function. In this case, it was reported that long intergenic noncoding RNA, named linc-RNA-ROR, regulates miR-145 which is crucial in regulating the self-renewal capability of stem cells as miR-145 directly targets the mRNA of TFs crucial for self-renewal such as the SOX2, Oct-4 and Nanog. As mentioned previously, the group also employed the use of reporter gene bounded to miR-145 target sequences. Luciferase gene fused with linc-RNA-ROR, Oct-4, SOX2 and Nanog transcripts, respectively, were transfected along with a miR-145 mimic (double-stranded RNA molecules that mimic miR-145 function). This resulted in the reduction in the reporter gene, Oct-4, SOX2 and Nanog activity, thus further confirming that miR-145 binds 3′-UTR of these genes [91,92].

MicroRNA sponges are more accurate in terms of defining specific miRNA targets since the target is cloned directly into a reporter gene mRNA. This enables the ectopic expression of plasmid sponges and miRNA mimic, which allows for more accurate results in miRNA sequence target confirmation in specific samples. On the other hand, expression of the target miR-sponges natively may result in the false reduction of reporter signal as endogenous miRNA may contribute to reporter silencing by competing for binding on the overexpressed target sequence. Additionally, the use of lentiviral plasmid allows for a more stable integration of the reporter system in cells which would result in a stable expression of the miR-targets [93,94].

However, there are cases of off-target effects brought about when using miR-sponges, this is because some miRNAs have similar seed sequences [95]. These seed sequences allow multiple miRNAs to bind to a similar target sequence. This could further complicate matters as targeted knockdown of a single miRNA may lead to the effect of targeting multiple miRNAs sharing similar seed sequences. Furthermore, miRNA sponges would compete with endogenous mRNAs with complementary 3′-UTR towards the target miRNAs this would result in steric hindrance, thus requiring more miR-sponges to suppress endogenous miRNAs. Although this might seem probable with titrations, too much sponge in itself may result in off-target effects [96,97].

### 3.2. Antisense Oligonucleotides/Antagomirs

Antisense oligonucleotides or generally known as antagomirs are a short sequence of RNA usually 13–25nt long [98]. Antagomirs are similar to siRNA tools as they bind target RNA to block their function [99,100,101]. These RNAs are complementary and can form base pairing with their target miRNA [102,103]. This would lead to the blockade of the miRNA function where miRNA in the miRISC complex cannot form complementary base pair with its target mRNA at the 3′-UTR. ASOs are often chemically modified by adding the 2′-*O*-methyl group to increase their potency [104]. Additionally, a pH-sensitive peptide carrier (pHLIP peptide) was also fused to ASO, this improves cell entry via the non-endocytic route [105,106].

miRNA control by ASO can be seen in research by Sun and colleagues, 2015, where the group silenced miR-320 [107]. The silencing of miR-320 led to the proliferation of glioma cells suggesting that miR-320 expression is crucial for the anti-proliferative effect. Additionally, the group also discovered that the E2F1 transcription factor, a target of miR-320, was upregulated.

ASOs were also employed in the silencing of miR-30a-5p in gliomas. In this study, it was found that the miRNA controls *SEPT7*crucial for proper neurogenesis and that overexpression of miR-30a-5p reduces the abundance of *SEPT7* leading to glioma development [108]. Additionally, ASOs was used in the treatment of leukemia where it targeted miR-126 which was found to be hyper expressed in patients with worse outcome. Targeting miR-126 resulted in the reduction of leukemic stem cells suggesting its critical role in leukemogenesis [109,110].

An advantage of using ASOs is that they are relatively easy to use; conceptually the ASOs will complementarily bind to its target sequence thus blocking the miRNA in the miR induced silencing complex (miRISC) from binding to its target mRNA. However, as simple as it may seem, the inhibition of target miRNAs requires the ASOs to be extensively modified [111]. Antagomirs can either be directly complementary or modified by adding modified ribonucleotides such as locked nucleic acid (LNA) a type of ribonucleotide with chemical bonds between the 2′oxygen and 4-carbon via a methylene bridge [112,113], ribonucleotides with phosphorothioate bonds [114] rather than phosphodiester bonds or nucleotide with modified 2′ position which can either be 2-O-methyl, 2-Omethoxyethyl or 2′-fluoro [115]. These modifications are crucial for the functioning of the antagomirs as the modifications would confer nuclease resistance and an increase in ASO potency. Generation of these modified ASOs involves complex chemical reactions despite being conceptually easy.

Furthermore, antagomirs usage interferes with downstream target mRNA quantification. Using qPCR-specific oligomers (primers or probes) designed against the target miRNAs, detection is based on the annealing of these oligomers onto the miRNA strand. Due to the formation of the ASO/miRNA heteroduplexes, these oligomers could compete for binding to the target miRNA resulting in a false positive reduction of miRNAs [116].

### 3.3. miRNA Mimic

**Figure 5:**
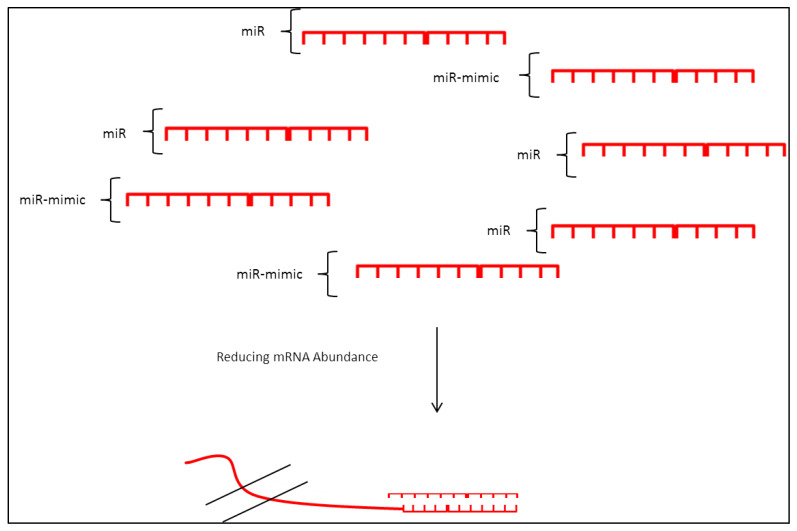
miRNA mimic uses short RNA sequence which mimics target miRNA bases sequence, this acts as a tool in inducing miRNA activity which would result in downregulation of target mRNA.

Mimics, as the name suggests, are double-stranded or single-stranded RNAs that have a similar sequence to miRNAs (Figure 5). These types of RNA act as an overexpression tool where they mimic the function of respective miRNAs [117]. Relatively easy to deliver and use, this technique becomes widespread in studying the effects of miRNA overabundance and miRNA replacement [118].

An example was recent research using mimics for miR-15a and miR16-1 in chronic lymphoid leukemia [119]. Results obtained showed that the two miRNAs affect CLL viability and that overexpression of these miRNAs using mimic significantly reduced cell viability suggesting that the use of these mimics could improve leukemic outcomes. Additionally, mimic for miR-494 targeting survivin, a crucial TF for the expression of kit in gastrointestinal stromal tumors, was also used [120]. Using 3′-UTR of survivin bounded to luciferase reporter gene the group co-transfected HeLa cells with miR-494 mimic and found that luciferase activity relative to negative control mimic was suppressed. Luciferase activity was rescued using inhibitors toward the miR-494 mimic.

A miRNA mimic was also used to study the effects of miR-126-5p/3p in AML as it was found to be highly expressed in patients with lower overall survival. Apoptosis inhibition and hyperproliferation were observed following mimic treatment suggesting miR-126-3p/5p is an oncomiR. This microRNA serves as a prognostic marker where overexpression represents a poor prognosis. This study also found that miR-126-3p inhibits cytarabine inducing apoptosis in a patient following chemo [121]. Here we can see the use of mimic in inducing specific miRNA expression without the use of lengthy and complex transduction or plasmid-based systems.

However, it was found that the use of this mimic would lead to passenger strand loading onto the miRISC complex leading to the unwanted effect of the passenger strands towards complementary 3′-UTR of genes [122]. This would lead to false-positive changes and the phenotypic changes may not be attributed to the miR-mimic overexpression. Another study also reported that post-mir-mimic transfection, cells tend to accumulate RNA at high levels [123]. This would lead to undesirable effects as it was found that the accumulation of high molecular weight RNA led to disease progression. Furthermore, the same study also reported that treatment of cells at a high concentration of miRNA-mimic would lead to non-specific changes in gene expression patterns. Surprisingly, they also found that mimic transfection leads to the accumulation of mutated endogenous miRNA and that this may result in non-specific gene modulation.

A summary of the various conventional techniques currently available to study miRNA function is depicted in Table 2.

## 4. CRISPR/Clustered Regularly Interspaced Short Palindromic Repeats as an Emerging Molecular Genetic Tool to Study miRNA Dysregulation

The previous sections of this review have shown that studying miRNA dysregulation is crucial in understanding cancer biogenesis and development. It is also apparent that an array of molecular techniques is readily available currently to elucidate miRNA function. The advantages and common pitfalls associated with each technique were described extensively as well. Additionally, the advent of genome editing has presented us with, yet another technique known as Clustered Regularly Interspaced Short Palindromic Repeat (CRISPR) to study miRNA dysregulation. The utilization of this versatile technique to elucidate miRNA function will be the focus of this review henceforth.

In essence, CRISPR or Clustered Regularly Interspaced Short Palindromic Repeats is a defense mechanism of bacteria from invading DNA-phage [132,133]. Briefly, it is a mechanism of acquired immunity in bacteria where the DNA of invading phages will be incorporated between the repeating spacer units that will be transcribed into the CRISPR RNA (crRNA) whereupon secondary phage invasion, this DNA will act as a guide in cleaving the invading phage via Cas9 endonuclease [134]. The guide RNAs are complementary to the viral DNA sequence, thus the directing of Cas9 on target viral DNA is based on RNA-DNA direct complementarity.

### 4.1. CRISPR/Cas9 (CRISPR Associated Protein 9)

CRISPR/Cas9 is a revolutionary tool used initially by Feng Zhang’s group to mediate genome engineering [135]. This technique employs the use of Cas9 endonuclease and small guide RNA (sgRNA) as tools for targeted genome editing. The sgRNA would form a complex with the Cas9 endonuclease. A twenty-nucleotide sequence within the sgRNA adjacent to the protospacer adjacent motif (PAM) sequence at the 5′ end of the sgRNA construct would then act as the homing beacon in defining specific target sequence via complementary base pairing.

The complementary binding defined by the sgRNA sequence would then bring the Cas9 towards the target genomic site and the endonuclease would subsequently cleave the double-stranded DNA and form a double-strand break (DSB). This would then initiate a non-homologous end joining (NHEJ) process where DNA repair machinery would start to reattach the breaks. This repair mechanism results in mutations such as insertion, deletion and random base substitution due to the fact that NHEJ is error-prone. The resulting mutation would render the target gene dysfunctional. The endonuclease capability of Cas9 also enables double nicking. This was observed in the Cas9 mutant termed Cas9-D10A, single-stranded DNA cleavage was observed within proximity of the PAM site. Using Cas9-D10A dual sgRNA system targetting the plus and the minus strand could be co-transfected with Cas9-D10A allowing for single-stranded nicking producing overhangs [136]. Reattachment of the overhangs would introduce indels via NHEJ.

Besides DSB, the foreign sequence could also be added into the genome via homology-directed repair (HDR) [137]. HDR is a mechanism where DNA repair machinery would recognize a foreign DNA strand that has the same sequence homology with the broken strand and integrate that foreign strand into the breaks (DSBs). Specific DNA sequence can be placed inside the strand in between 5′ end and 3′end homology sequence where it can be incorporated into the repaired strand through the pairing of the introduced strand to the broken strand via complementary base pair of the homology sequence where subsequently the foreign strand will be ligated into the broken strand.

Utilizing the principle mentioned above (CRISPR/Cas9), one could either increase miRNA expression (HDR), do a knock-in study (HDR) or knockout the miRNA gene (NHEJ) directly on the genome level as this technique allows for highly specific genome editing. However, there are consequences of off target effects where the endonuclease would cleave non-targeted genomic loci. Various other CRISPR methods are available to mitigate this bottleneck and will be described subsequently.

#### 4.1.1. CRISPR/Cas9 Direct Modulation of miRNA Genome

Several studies have shown the use of CRISPR in directly modulating miRNA genes [138,139]. This was shown to be effective in downregulating the expression of miR-17 [140]. Xi and group showed that designing small guide RNA targeting the 5′p and 3′p region of the pre-miRNA containing the dicer and drosha processing sites resulted in a significant reduction in the levels of mature miRNA expression and upregulation of the primary miRNA transcript. Additionally, it was also found that E2F1, a target of miR-17, was upregulated. PCR and subsequent sequencing of cleaved genomic target reveal different mutations which included deletions and insertions that were brought about by NHEJ on the miRNA loci.

Additionally, CRISPR/Cas9 was also used in depleting miR-210-3p in renal cell carcinoma [141]. Depletion targets the 3′p seed sequence and the stem-loop sequence of pre-miRNA-210-3p where Cas9 endonuclease cleavage resulted in the reduction of the mature miR-210-3p further showing that the CRISPR/Cas9 system can be used to directly target the genomic loci in which the miRNA gene is present.

However, certain complications could arise from using this technique since Cas9 cleavage results in indels due to the double-strand breaks generated. A common pitfall in using this technique is that if the PAM sequences are located inside the miRNA seed sequence, Cas9 cleavage could result in a mutant miRNA due to indels. Processing of the primary transcript would result in mutant miRNAs that target different genes and cause changes in phenotype, producing false-positive results. This is highly plausible as it was reported that a difference of four nucleotide bases could potentially cause off-target binding of sgRNA targeting for different miRNAs [140].

#### 4.1.2. Cas9 Modulation: miRNA Gene Expression Control

Cas9 endonuclease cleavage of genomic loci is defined by the sgRNA. As such, the docking of Cas9 on a specific genome locus is based on sequence complementarity of the sgRNA and DNA forming the sgRNA-gDNA hybrid. This has allowed scientists to direct certain functional protein domains towards targeted gene loci via fusion with Cas9 to induce specific changes in gene expression [142,143,144]. This proof of concept was initially reported by Gersbach’s group in which they mutated the D10A mutant of Cas9 via site-directed mutagenesis and fused the “dead” Cas9 termed dCas9 to VP-64 a functional activator of gene expression [145]. The group also designed various enhancements on the dCas9 allowing for epigenetic modulation, gene repression, cell line-specific reprogramming, epigenetic reprogramming towards specific cells lines and endogenous gene expression control [146,147,148,149].

Using the dCas9-VP-64, Gersbach and colleagues designed sgRNAs targeting the promoter of *IL1RN*. Plasmid cassettes containing the dCas9 and sgRNA were then transfected into HEK293T. RNAseq and sub sequential DESEq analyses revealed only upregulation of *IL1RN* thus suggesting that the technique is highly specific. The research was also carried out by targeting the promoters of *NANOG*, *MYOD, TERT*, *VEGFA*, *IL1B* and *HBG1* and *2*. The results show significant upregulations of these genes. They also reported that binding of the dCas9-activator complex to enhancers and distal promoters could also activate gene expression [145].

Owing to the fact that crRNA-guided gene activation is specific as dCas9 could carry specific transactivation domains, Gersbach and colleagues also fused dCas9 with deactivators [150]. In this case, KRAB (Krüppel-associated box) domain was utilized to target a distal enhancer of the *HBG* gene. Results showed a significant reduction in *HBG1* and *HBG2* expression and an increase in heterochromatin signature indicating repression in the enhancer site. This interferes with the binding of native transcription factor onto the targeted loci bounded by dCas9-KRAB, leading to gene repression.

Using the same concept; theoretically, we can apply these techniques in inducing or repressing miRNA. This can be carried out by designing the sgRNA to be specific against a miRNA locus. Using the dCas9-VP64 activation tool, Gersbach induces expression of the targeted gene via multiple sgRNA transfections. This allows multiple binding of the fusion product to a proximal sequence within the promoter which will increase the VP-64 docking via dCas9 onto gene promoters resulting in a significant increase in gene expression. This could be employed by using plasmid cassettes which allow for the expression of multiple sgRNAs. Ventura’s lab utilized pX33 (addgene) specifically for in vivo application this plasmid enabled multiple sgRNA expression. This significantly increased VP-64 docking onto the miRNA gene locus thus activating them [151]. On the other hand, pX330A-1X6 (Addgene) from Yamamoto’s lab can be employed for in vitro applications. This plasmid allows for the expression of up to seven different sgRNAs which enables multiple dCas9-VP64 docking onto target sites which would increase miRNA expression. This is especially useful for a gain of function study [152]. However, for efficient gene induction, VP-64 must be bounded onto the gene locus at higher copy numbers. This could increase the chances of off-target due to the introduction of multiple sgRNAs.

Additionally, miRNA expression could also be repressed via dCas9-KRAB and sgRNA that targets specific miRNA locus. In this case, the KRAB domain induces epigenetic changes leading to heterochromatin by inducing histone trimethylation. This will result in a “tight” coil formation of DNA surrounding histones which would impair the accessibility of crucial transcription machinery onto their respective binding sites thus suppressing gene expression [153]. Owing to this fact, KRAB could be directed to specific miRNA genomic locus where the formation of heterochromatin would impair crucial transcription machinery docking onto miRNA locus thus suppressing miRNA expression [154,155]. The plasmid cassette that can for utilized for this purpose is the pLV hU6-sgRNA hUbC-dCas9-KRAB-T2a-Puro (Addgene) from the Gersbach lab. This plasmid could be used to co-express sgRNA along with dCas9-KRAB [150].

Proof of concept in the utilization of dCas9 for miRNA targeting was carried out by Zhao and colleagues where they designed sgRNA targeting the 5′ upstream sequence of miR-17-92 cluster loci [156]. This results in the reduction of mature miR19a, miR20 and miR-92 expression suggesting that dCas9 could be used to block the binding of crucial TFs thus blocking transcription elongation of pre-miRNA transcript. Therefore, using dCas9 alone without KRAB may suppress miRNA expression. However, it is highly probable that epigenetic modulation brought about by the KRAB domain would improve the efficiency of knockdown [157].

### 4.2. CRISPR/Cpf1 (CRISPR Associated Endonuclease in Prevotella and Francisella 1)

CRISPR/Cpf1 is a type II system that targets specific cleavage via RNA-guided docking of Cpf1 instead of Cas9 [158]. The Cpf1 system is more straightforward as compared to the Cas9 system in principle. Cpf1 1 requires a crRNA of approximately 42 nucleotides in length only and no complexing with tracrRNA is required. Furthermore, it was found that plasmid constructs containing only Cpf1 and sgRNA sequences are enough to induce DNA cleavage, thus making this system relatively easier than Cas9. However, the cleavage site of Cpf1 is located further away from the PAM site.

The advantage of using this system as compared to the Cas9 system is the generation of sticky ends, which would facilitate NHEJ mediated gene insertion. Since Cpf1 endonuclease activity resulted in 5bp overhangs, double-stranded DNA sequence carrying desired genes with flanking complementary sites towards the 5bp overhangs would improve gene knock-in via base pairing between the 5bp overhangs and gene inserts [159,160]. However, the suppression of microRNA demands more technical procedures as Cpf1 delivery would require both the endonuclease and the sgRNA to be delivered simultaneously. This can be observed in the lentiviral delivery of the Cpf1 system where plasmids carrying the endonuclease and the sgRNA would require genomic integration for stable gene knockout as in Cas9. Therefore, this approach could be technically more demanding than the delivery of antagomirs. This system also comes with an activator, however, it is currently used to target endogenous genes [161,162]. No direct targeting of miRNA by Cpf1 was reported to date, however, with the availability of Cpf1 tools for activation, we could induce acetylation of histones on miRNA locus activating miRNA expression as miRNA promoter was found to be associated with high histone acetylation state [163]. Thus far, using Cpf1 against miRNA is still lacking due to Cas9 still being the golden rule. However, the use of Cpf1 to suppress miRNA could be a potential field for applications development.

### 4.3. Cas12b/C2C1

Similar to Cpf1, Cas12b contains a single nuclease domain the RuvC domain making it smaller than Cas9. However, Cas12b requires tracrRNA to be incorporated for genomic cleavage this mechanism is similar to that of Cas9. PAM site for Cas12b is TTN compared to Cpf1 TTT. Engineering of the C2C1 by substituting amino acid residues at three different sites on the RuvC domain confers higher genomic cleavage efficiency and higher specificity as the percentage of perfect edits was found to be higher than that of Cas9 [164].

This Cas subtype may be more suitable in miRNA targeting as it has higher edit specificity. This should reduce the formation of off-target miRNAs resulting from Cas genomic cleavage.

### 4.4. CRISPR/Cas13

Cas13 or originally known as C2C2 was found to mediate direct RNA cleavage. There are various subfamilies of Cas13 consisting of Cas13a, Cas13b, Cas13c and Cas13d [165]. Cas13 endoribonuclease activity does not require PAM sequences and it was reported that direct complementarity towards target sequences via RNA-RNA hybrid is sufficient [166,167]. Furthermore, a small guide RNA sequence of 28nucleotide with flanking 36 nucleotide spacer was found to be sufficient for Cas13 mediated cleavage of mRNA where efficiency was comparable to that of shRNA mediated targeting with much higher specificity as shown by RNAseq data [167]. Although efficient, this technique requires screening of the small guide RNA as studies have shown that guides targeting a different region of RNA resulted in a different suppression efficiency, thus being more time-consuming.

Additionally, it was also shown that Cas13 could mediate RNA editing. It was shown that by fusing the dCas13 with an enzyme named adenosine deaminase acting on RNA (ADAR), the base substitution of adenosine to inosine (G) in RNA transcripts, could be performed [166]. This base substitution would reconstitute the mRNA sequence thus affecting translation.

Using these systems, miRNA could either be cleaved or edited. For example, in cases such as a point mutation occurring in a miRNA gene sequence, the mutation could be edited via dCas13. The repaired miRNA could resume its normal function as a single point change in the seed sequence would hamper miRNA function [81,168]. Additionally, Cas13 could also be used to down-regulate specific miRNA activity by designing the small guide sequence to be complementary towards the target miRNA. Currently, Cas13 has the potential to target miRNA but research still lacks in this field. This opens up a great opportunity for Cas13 research into miRNA targeting.

### 4.5. Utilization of CRISPR to Target miRNA: Practical Considerations

The previous section of this review has shown that arrays of CRISPR techniques are available for miRNA targeting. In view of this, some important considerations which could serve as a general guideline will be highlighted shortly.

**Figure 6:**
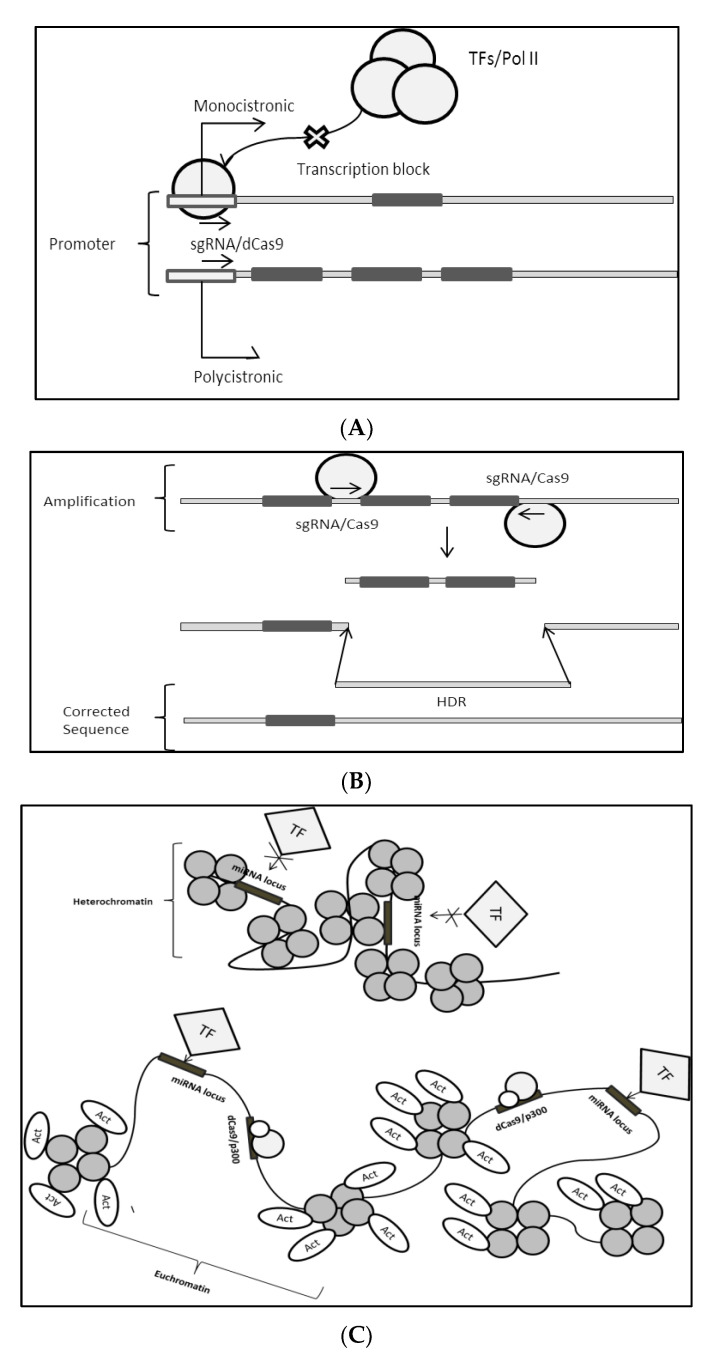

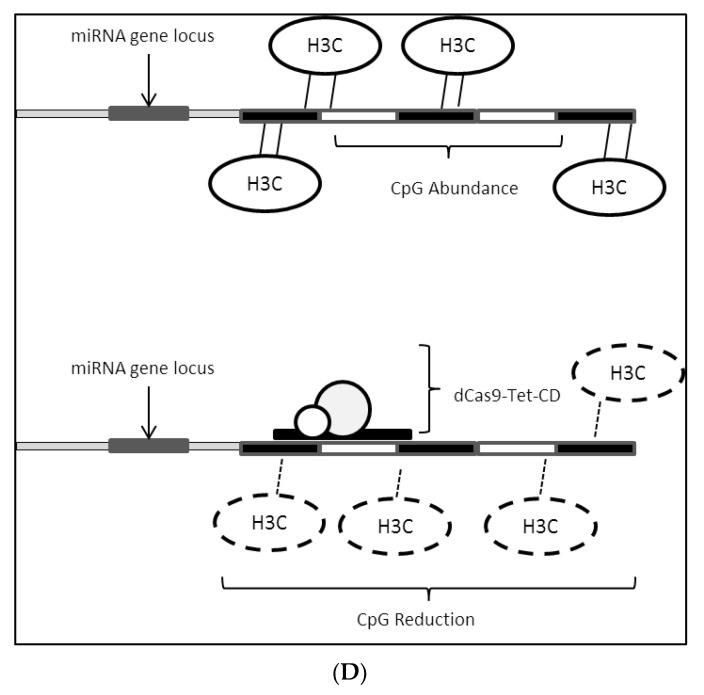
(**A**) Using dCas9 to block transcription elongation. TFs: transcription factors; Pol II: RNA polymerase 2; (**B**) Using the nickase strategy to cut amplified loci. HDR: homology-directed repair; (**C**) Relieving the epigenetics state of miRNA locus to increase gene transcription. Act: Acetyl group; p300: Histone modifier; (**D**) Modulation of the CpG methylation to induce miRNA expression. Modulation of the CpG methylation to induce miRNA expression. H3C: methyl group.

Few strategies could be employed depending on desired outcomes. miRNA for example could be indirectly targeted by designing CRISPR against their activators, transcription factors responsible for driving their expression, as in the case of c-MYC [169]. This could repress the expression of targeted miRNA via transcriptional block. In contrast, suppressors could also be targeted in order to induce the expression of a targeted miRNA. However, targeting TFs would induce a global expression change as these TFs could also bind to other promoters containing its consensus binding sequence, thus it is not advisable to do this (Figure 6A).

Apparently, this could be mitigated by targeting a specific site by engineering sgRNA sequence directing dCas9 towards that particular site. This would not cause a change in global expression pattern as the dCas9 blocks TFs from actually binding onto their consensus sequence found only on the miRNA locus, thus achieving a specific miRNA knockdown. It should also be understood that these sequences are promoters/enhancers and that promoters could either be polycistronic or monocistronic [170,171]. If a particular miRNA locus is driven by only one promoter doing this is favorable, if it is polycistronic where a particular promoter can actually drive multiple miRNAs or even genes within close proximity to the miRNA, this technique becomes unfeasible.

In the case where miRNA overexpression is caused by the amplification of loci, the Cas9 nickase strategy could be employed. This could be achieved by designing two sgRNAs that complement flanking amplified sites, where it would result in “nicking” or removal of a fragment of targeted loci [172,173] (Figure 6B). Amplified fragments could be removed using this method. However, miRNA are short sequences of RNA and that only the seed sequences are responsible for complementing target mRNA therefore, removal of large loci is not advisable. Additionally, via homology-directed repair (HDR), a fragment with the “correct” sequence could be re-introduced to restore the “nicked” fragment of the genome. This is crucial as to not alter the sequence which may be crucial for other cellular gene activity.

Epigenetics plays an important role in the expression of miRNA, chromatin state brought about by histone deacetylase (HDAC) and acetyl transferase (HAT) plays a huge role in allowing TF accessibility onto a genomic region that drives the expression of that particular miRNA. Euchromatin state in heterochromatin can be induced by bringing the p300 close to the region of interest that could be in supercoiled state (heterochromatin) [174,175] (Figure 6C). p300 is an activator domain that acts as histone acetyl transferase, where it catalyzes the transfer of acetyl group onto histones. This process relaxes the DNA-Histone coil allowing the region targeted with p300 to be more accessible towards the TF/pol II complex which would result in an increase in transcriptional activity. This is especially helpful when applied to activate downregulated miRNA crucial for proper development. Additionally, if a particular miRNA is over-expressed, dCas9-KRAB is useful where it helps in forming heterochromatin on the highly accessible miRNA loci as explained earlier [150]. Therefore, it is crucial to identify the type of dysregulation occurring on the miRNA loci before proceeding with this method. One way of doing so is to identify enrichment of histone states via ChIP-qPCR (for a specific site) or ChIP-Seq (for global enrichment of histone states) [176].

An overabundance of CpG islands often led to gene silencing; using Tet-CD (Ten eleven translocationcatalytic domain) this could be alleviated via its demethylase activity. Tet-CD is a functional domain of a demethylase responsible for oxidizing the methyl on cytosine (Figure 6D). This helps in reducing DNA methylation which would promote gene activation. It was clearly shown that the abundance of CpG islands on miRNA loci would impair expression resulting in the downregulation of particular miRNAs crucial for proper development. Therefore, via CRISPR, Tet-CD fused to dCas9 could be used to specifically target Tet-CD onto the CpG rich sites allowing for demethylation of target sequence thus restoring miRNA expression adjacent to the targeted site [177,178]. CpG abundance across a particular miRNA genome can be identified by bisulfide sequencing which measures relative levels of methylation across loci [179].

A summary of the various CRISPR based techniques used to study miRNA dysregulation in cancer is depicted in Table 3. Table 3: Various CRISPR based techniques that are used to study miRNA function in cancer.

## 5. Conclusions: Challenges and Future Perspectives

**Figure 7:**
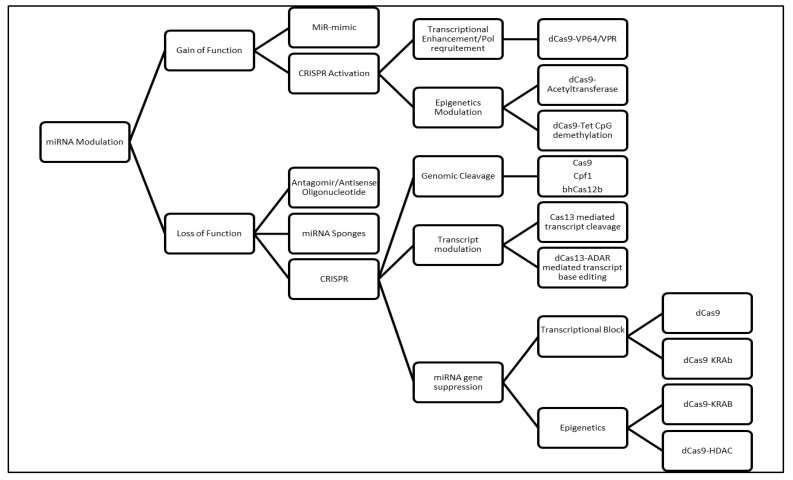
General workflow for studying miRNA dysfunction in cancer. Depending on experimental design miRNA study could be assessed via knock-in or loss of function study. Several CRISPR-based methods are available as listed in Figure for the desired experiments.

It is clearly apparent that studying miRNA dysregulation in cancer is vital for us to understand cancer biology. A wide array of molecular biology techniques are readily accessible for the researcher to conduct the relevant experiments. Nevertheless, extreme care is needed to choose the correct molecular biology tool in order to answer the relevant research questions in regard to miRNA dysregulation in cancer. A general workflow is provided in Figure 7.

Most researchers will find it tempting to utilize CRISPR/Cas-based techniques for studying miRNA dysfunction due to their specificity and exquisiteness. However, there are certain issues and challenges that should be taken into account when employing these techniques in miRNA targeting. This is mainly due to the complexity of miRNA regulation. Points to consider include miRNA location, distribution and targets. This was exemplified by the miR-17-92 cluster which was implicated in many cancers [189]. However, the miR-17-92 is a cluster where different miRNAs reside proximally between one another; this will prove to be a challenge when using Cas9, as indels may induce mutation into the miRNA sequences resulting in either miRNA that is null or off-target miRNA. Additionally, clusters of miRNA are usually polycistronic, where a single promoter drives the expression of multiple miRNAs. Therefore, targeting a single miRNA in a cluster would be a challenge since this would affect adjacent miRNAs.

Micro RNA that resides within exonic sequence would also hamper targeting via CRISPR. Targeting these miRs may result in disruption of the splicing machinery leading to changes in the mRNA component of the host gene. This is a major hole in miR targeting using CRISPR. Even if there is an argument of using dCas9 for the transcriptional block instead of genome cutting considerations on how the mechanism induces silencing must be taken. Dead Cas9 blocks transcription elongation, targeting miRNA located in the host gene. Using this method is not advised as the experimenter must consider the simultaneous silencing of both target miR and host gene.

miRNA seed sequence targeting complexity of genes would also prove to be a challenge in direct targeting. In breast cancer (BC) it was found that miR-139-5p targets multiple genes involved in crucial BC oncogenesis [190]. Even with CRISPR/Cas-mediated suppression of miR, phenotypic changes would not be induced by a single gene expressional change. This requires further screening of the genes which would be time-consuming and costly. Furthermore, direct miRNA targeting is not as straightforward as targeting a different region of the miR transcript would result in a different suppression efficiency [191].

In conclusion, it can be said that CRISPR/Cas 9-based tools will gradually replace other conventional methods in studying miRNA dysregulation. Nevertheless, the relevance of more conventional techniques such as miRNA sponges, antagomirs and miRNA mimic cannot be negated altogether as these tools could prove to be extremely useful, especially when the specific miRNA is not pliable to CRISPR/Cas-based genetic manipulation.

## Figures and Tables

**Table 1:** Table showing various mechanisms of dysregulation of miRNA in cancers.

miRNA	Mechanism of Dysregulation	Consequence	References
miR-650	Loci amplification	Inverse correlation was observed between miR-650 and tumour supressor genes ING4 and NDRG2	[77]
miR-21	Loci amplification	miR-21 overexpression leads to PTEN suppression	[78]
miR-4288	Deletion	Loss of miRNA in prostate cancer, miRNA directly represses metastatic/invasion genes MMP16 and ROCK1	[79]
miR-3613	Deletion	miR-3613 was found to be lower in breast cancer. Gain of function reveals miR-3613 to regulate PAFAH1B2 and PDK3 blocking oncogenesis	[80]
miR-379	Base substitution	Hotspot mutation commonly occurring in lung adenocarcinoma	[81]
miR-142-3p	Epigenetic suppression via DNMT recruitment	Hypermethylation of miR-142 leads to unfavorable prognosis in nasopharyngeal carcinoma	[82]
miR-338-5p/421	EZH2 mediated suppression via DNA methylation	Presence of CpG marks on primary prostate cancer. Ectopic expression reveals suppression in prostate cancer growth	[83]
miR-17-92/106b-25	CMYC driven	CMYC drive the expression of these miRNA clusters, inhibition of cMYC activators resulted in suppression of these clusters in hepatocellular carcinoma	[84]
miR-122	CMYC driven	CMYC oncogene overexpression in hepatocellular carcinoma activates miR-122 via direct promoter binding driving oncogenesis	[85]
miR-455-3p	Reside in host gene driven by p53	miRNA involves in cancer quiescence via p53 mediation	[86]

**Table 2:** Various conventional techniques to study miRNA function.

Method of Study	miRNA	Mechanism	Outcome	References
miRNA sponges	miR-21	Synthetic RNA sponge was designed to bear sequence complementary to miR-21	Downregulated proteins due to miR-21 overexpression were restored	[124]
	miR-21 and miR-93	Single RNA sponges bearing multiple complementary sites against target miRNAs	Targetting oncomiRs effectively induce apoptosis and blocked proliferation of esophageal carcinoma	[125]
	miR-223	RNA sponge Expressing DNA plasmid was used. RNA circularization was imposed using slicing acceptor and donor site	Sponges effectively sequester endogenous miRNA in T-ALL cells effective restoration of miR target genes	[126]
Antagomirs/Antisense Oligo nucleotide	miR-155-5p	Transfection of breast cancer cell lines with antagonist against miR	Downregulation of miR leads to increase in breast cancer sensitivity against cetuximab	[127]
	miR-125a-5p	Transfection of gastric cancer cells with antagomir	Restoration of miR suppressed genes was observed, suppression leads to the suppression in EMT of gastric cancer	[128]
	miR-155	Delivery of antagomir into MCF-7 via attachment to gold nano particle	Elevation in miR target gene T53INP1 was observed stimulating apoptosis of breast cancer cells	[129]
miRNA mimics	miR-27a	Overexpression of miR via mimics transfection Mimics used are as standard siRNA sizes	Overexpression of miR-27a via mimic alleviates cancer characteristics and sensitizes breast cancer towards anticancer drugs	[130]
	miR-150	miRNA sequence was expressed using pEZX-MR via lentiviral delivery	Mimic expression induces apoptosis in multiple leukemic cell lines	[131]

**Table 3:** Table showing miRNA target using CRISPR/Cas9 via different modes at miRNA processing stage.

microRNA Target	Target Site	CRISPR System, Delivery	Model	Outcome
miR-93 [180]	5′ Drosha Processing site	CRISPR/Cas9, Lipofection	Human Cervical Cancer (HeLa)	Almost no detection of mature miR-93 Accumulation of primary miR-93 transcript suggest impairment in Drosha processing
miR-21 [181]	20nt sequence adjacent to PAM (NGG)	CRISPR/Cas9, Lentiviral Vector	Human Ovarian Adenocarcinoma (SKOV3 and OVCR3)	Significant reduction in mature miR-21 expression was observed
miR-130a [182]	5p and 3p Seed Sequence Stem Loop (Dicer binding Site)	CRISPR/Cas9, Lipofection	Human Breast Cancer (MCF7)	Significant reduction was observed when using Cas9 targeting from the 5p region No significant difference in miR-130a expression was observed when targeting either the 3p or the Stem Loop sequence
miR-137 [183,184]	Nucleotide sequence upstream of 5′ PAM (NGG)	CRISPR/Cas9, Lentiviral Vector	Human Ovarian Carcinoma (A2780)	Significant reduction in mature miR-137 expression was observed. Deletion and insertion mutation detected from single-cell expanded colonies.
miR-379/miR-656 cluster [185]	dCas9 fused to VP-64 docking on the miRNA locus for induction of miRNA gene expression.	CRISPR/dCas9, Lipofection	Human Glioblastoma	Increase in expression of miRNA within the miR-379/miR-656 cluster post-dCas9-VP64 gene induction.
miR-23b and miR-27b [186]	Annotated Stem-loop region	Cas9/Lentiviral Transduction	Human Breast Cancer (MCF7)	Significant reduction of miR-23b and miR-27b transcripts was observed
miR-423 [187]	miR-423 locus	Cas9/Lipofection	Human Cervical Cancer (HeLa)	Significant knockdown of miR-423 transcripts was observed
miR-17-92 [188]	miR-17-92 5p loop	CRISPR/Cas9 nickases	Anaplastic Thyroid Cancer	Knockdown of clusters was observed

## Data Availability

Not applicable.
